# Pyorrhea Alveolaris

**Published:** 1913-09-15

**Authors:** E. A. Lundy

**Affiliations:** Los Angeles


### PYORRHEA ALVEOLARIS.

BY DR. E. A. LUNDY, LOS ANGELES.

As the majority of the papers published on this subject have been on the etiology of this disease, I have thought it best to prepare a paper on the practical requirements necessary for the successful treatment of this dire disease which has become more prevailent from year to year as people have become more intense in their desire to obtain the almighty dollar, and the luxurious living attendant with the acquirement thereof.

The following are some of the requisites to a succesful issue in the treatment of this disease: a dogged tenacity of purpose, a thoroughness in every detail, a sensitive and delicate touch, and a mature judgment of the requirements of each case. In all aggravated cases it is my practice to first administer a palliative treatment to relieve the congestion present and to inhibit pus formation.

All teeth that are unfit for retention are removed, all malocclusion is reduced and a most thorough and careful instrumentation is given to each tooth. All necrotic process is curetted, and this feature requires the utmost care and judgment to the end that it should be done thoroughly, and yet not overdone to the extent of producing thrombosis.

I find it better to use an anesthetic, as by this means the work is rendered less arduous to the operator and more bearable to the patient.

The instrumentation should be most careful and thorough, without unnecessary manipulation, to the end that the cementum be not removed or made so thin that pulp irritation results. I have seen cases where the cementum had been planed away until the teeth were so sensitive that the only permanent relief was the removal of the pulp.

There are some other details that should be considered such as the avoidance of extra manipulation; in all deep pockets one should place the instrument with deliberation, and keep it in the pocket until all deposits detected are loosened; the angle of the handle should also be carefully noted to avoid unnecessary impingement on the vital tissues. To avoid confusion and to economize time a multiplicity of instruments should be avoided.

It is my practice after the careful removal of deposits and the polishing of the teeth to wash the pocket with a mild antiseptic and stimulant and to apply an antiphlogistic medication to the gum. I also close all open pockets with an antiseptic packing so that the impaction of food is thereby avoided and the tissues allowed to fill in from the bottom of the pocket, I find it advisable to avoid the use of irritating and escharotic remedies, which handicap rather than help the healing process. With my methods of treatment patients are able to masticate comfortably in a very short time.

Massaging of the gum is beneficial, and it is likewise essential that the teeth be kept thoroughly cleansed and polished. All impingement on the gum tissue in the interproximal spaces should be avoided and all fillings and crowns so placed as to avoid impaction of food. All loose teeth should be given proper support, and those that have but a small pericemental attachment should be permanently supported.

In the opinion of many dentists who are specializing in the treatment of pyorrhea, this is a disease caused simply by uncleanliness, or by irregularity and malocclusion and amenable to local treatment only. Now this may obtain in a minority of cases, but in my opinion gleaned from twenty-five years of experience in the treatment of this disease, the majority of cases are simply an oral manifestation of a systemic disturbance. For twelve years past my treatment has been both from a local and systemic standpoint and the results obtained have been far more satisfactory than formerly. I have embraced this view in direct opposition to the teachings of some prominent dental educators who still adhere to the local treatment and consider it amply sufficient. On the other hand, there are just as many who adhere to the duality of systemic and local causation.

My systemic treatment varies with individual cases, but is arranged with a view to the establishment of normal elimination and assimilation. I find in the majority of cases that constipation is present with resultant autointoxication, and my treatment aims at the overcoming of these conditions. My efforts may be at first in the line of internal medication, but later I resort to a proper dietary, suggesting suitable foods which require chewing. I also prescribe a non-uric dietary.

My favorite remedies for local treatment at present are Dr. Senn's solution of iodin and potassium iodid, one part to water 100 parts, making practically a one percent solution This preparation, which I use in the pockets, is supplemented with stimulating applications on the gingival mucous membrane.—*Pazific Gazette,* August, 1912.

